# Subclinical Myocardial Dysfunction in Patients with Persistent Dyspnea One Year after COVID-19

**DOI:** 10.3390/diagnostics12010057

**Published:** 2021-12-28

**Authors:** Maria-Luiza Luchian, Andreea Motoc, Stijn Lochy, Julien Magne, Dries Belsack, Johan De Mey, Bram Roosens, Karen Van den Bussche, Sven Boeckstaens, Hadischat Chameleva, Jolien Geers, Laura Houard, Tom De Potter, Sabine Allard, Caroline Weytjens, Steven Droogmans, Bernard Cosyns

**Affiliations:** 1 Department of Cardiology, (Centrum voor Hart-en Vaatziekten), Universitair Ziekenhuis Brussel, Vrije Universiteit Brussel (VUB), 1090 Brussels, Belgium; andreeaiulia.motoc@uzbrussel.be (A.M.); stijn.lochy@uzbrussel.be (S.L.); bram.roosens@uzbrussel.be (B.R.); karen.vandenbussche@uzbrussel.be (K.V.d.B.); sven.boeckstaens@uzbrussel.be (S.B.); Jolien.Geers@uzbrussel.be (J.G.); laura.houard@uzbrussel.be (L.H.); caroline.weytjens@uzbrussel.be (C.W.); steven.droogmans@uzbrussel.be (S.D.); bcosyns@gmail.com (B.C.); 2 Service Cardiologie, CHU Limoges, Hôpital Dupuytren, 87000 Limoges, France; julien.magne@unilim.fr; 3 16 INSERM 1094 Faculté de Médecine de Limoges, 87000 Limoges, France; 4 Department of Radiology, Universitair Ziekenhuis Brussel, Vrije Universiteit Brussel (VUB), 1090 Brussels, Belgium; dries.belsack@uzbrussel.be (D.B.); johan.demey@uzbrussel.be (J.D.M.); 5 Faculty of Medicine and Pharmacy, Vrije Universiteit Brussel (VUB), 1090 Brussels, Belgium; Hadischat.Chameleva@uzbrussel.be (H.C.); tom.de.potter@vub.be (T.D.P.); 6 Department of Internal Medicine, Universitair Ziekenhuis Brussel, Vrije Universiteit Brussel (VUB), 1090 Brussels, Belgium; sabine.allard@uzbrussel.be

**Keywords:** long COVID-19, echocardiography, myocardial work, global longitudinal strain, persistent dyspnea, subclinical dysfunction

## Abstract

Long coronavirus disease 2019 (COVID-19) was described in patients recovering from COVID-19, with dyspnea being a frequent symptom. Data regarding the potential mechanisms of long COVID remain scarce. We investigated the presence of subclinical cardiac dysfunction, assessed by transthoracic echocardiography (TTE), in recovered COVID-19 patients with or without dyspnea, after exclusion of previous cardiopulmonary diseases. A total of 310 consecutive COVID-19 patients were prospectively included. Of those, 66 patients (mean age 51.3 ± 11.1 years, almost 60% males) without known cardiopulmonary diseases underwent one-year follow-up consisting of clinical evaluation, spirometry, chest computed tomography, and TTE. From there, 23 (34.8%) patients reported dyspnea. Left ventricle (LV) ejection fraction was not significantly different between patients with or without dyspnea (55.7 ± 4.6 versus (vs.) 57.6 ± 4.5, *p* = 0.131). Patients with dyspnea presented lower LV global longitudinal strain, global constructive work (GCW), and global work index (GWI) compared to asymptomatic patients (−19.9 ± 2.1 vs. −21.3 ± 2.3 *p* = 0.039; 2183.7 ± 487.9 vs. 2483.1 ± 422.4, *p* = 0.024; 1960.0 ± 396.2 vs. 2221.1 ± 407.9, *p* = 0.030). GCW and GWI were inversely and independently associated with dyspnea (*p* = 0.035, OR 0.998, 95% CI 0.997–1.000; *p* = 0.040, OR 0.998, 95% CI 0.997–1.000). Persistent dyspnea one-year after COVID-19 was present in more than a third of the recovered patients. GCW and GWI were the only echocardiographic parameters independently associated with symptoms, suggesting a decrease in myocardial performance and subclinical cardiac dysfunction.

## 1. Introduction

Coronavirus disease 2019 (COVID-19), caused by severe acute respiratory syndrome coronavirus 2 (SARS-CoV-2), rapidly spread across the globe with more than 260 million cases worldwide, overwhelming the healthcare systems [1,2,3].

Clinical manifestations occur predominantly due to lung involvement, however, there is a growing body of evidence showing the presence of cardiovascular complications attributed to SARS-CoV2 infection [4,5].

COVID-19 pneumonia is characterized by a wide spectrum of symptoms including fever, cough, dyspnea or chest pain as well as headache, ageusia or anosmia [6,7]. Advanced age and a history of cardiovascular or respiratory diseases are well established risk factors for a more severe course of COVID-19 [2,8]. Although it is considered primarily a respiratory disease, several other organs may be involved via angiotensin converting enzyme 2 (ACE2) receptors which are also located at the level of the endothelial cells, explaining the complexity of symptoms and complications including myocardial injury [9,10].

In clinical practice, earlier reports showed an increased prevalence of high cardiac troponin levels, a surrogate biomarker for myocardial injury, which was further associated with impaired left ventricular relaxation and reduced right ventricle function leading to higher morbidity and mortality rates, including possible long-term consequences [11,12,13,14,15].

Following an acute episode of COVID-19, several short-term follow-up studies emphasized the persistence of symptoms, referred as long COVID, in a significant number of discharged patients even without a history of cardiopulmonary diseases, with dyspnea being one of the most frequent complaints [16]. Although in young and apparently healthy adults, SARS-CoV-2 infection is mostly mild and not requiring hospitalization [17,18], data on the presence of cardiac sequelae due to COVID-19 remain contradictory.

Therefore, considering the importance of cardiac screening using multimodality imaging, for instance, in sports competitions [19,20], several reports addressed the presence of potential cardiac damage after an acute COVID-19 episode in young competitive adults. Cardiac involvement was commonly described at two- to six-month follow-up [17,18,21,22,23], hence emphasizing the necessity for long-term cardiac surveillance.

Even though those reports on recovered COVID-19 patients did not describe major abnormalities of the left ventricle (LV) function [24,25], subtle cardiac changes attributed to SARS-CoV-2 infection cannot be entirely dismissed.

Moreover, previous COVID-19 follow-up studies remain limited by the short-term follow-up duration and the heterogeneity of the population. Therefore, we sought to investigate the presence of subclinical cardiac dysfunction, assessed by transthoracic echocardiography (TTE), in recovered COVID-19 patients without previous cardiopulmonary diseases at one-year follow-up.

## 2. Materials and Methods

A total of 310 consecutive hospitalized patients with confirmed COVID-19 infection by real-time reverse transcription polymerase chain reaction were prospectively included between March and April 2020. Sixty-six patients out of 251 recovered patients had no previous history of coronary artery disease, arrhythmia, arterial hypertension, valvular heart disease, asthma, chronic obstructive pulmonary disease and obstructive sleep apnea, respectively, and were included in the final analysis. The follow-up consisted in two parts, a six-months visit including clinical and physical examination, chest computer tomography (CT), and spirometry and a 12-months visit including clinical and physical examination, spirometry, and TTE.

**Figure 1:**
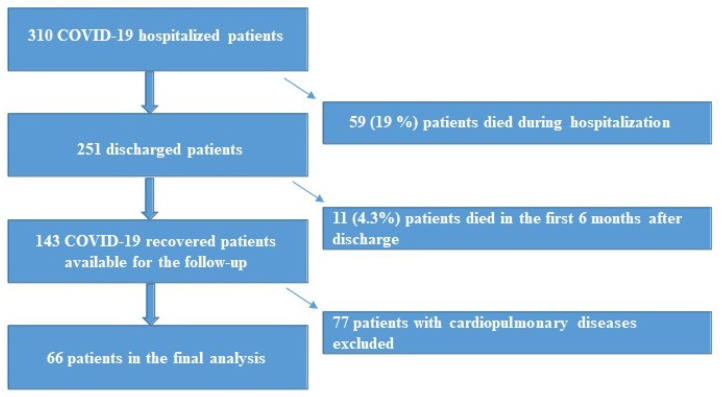
Schematic representation of the study population.

The study design is summarized in Figure 1.

### 2.1. TTE

All the patients underwent a comprehensive TTE (GE, Vivid E9, Vingmed Ultrasound, Horten, Norway). M-Mode, speckle tracking and myocardial work, two-dimensional (2D), and Doppler measurements were performed following the standard recommendations using GE Healthcare, Vingmed Ultrasound, Horten, Norway, EchoPAC version 20.3. [26,27].

### 2.2. Speckle Tracking Echocardiography (STE)

2D apical four chamber, two chamber and apical long axis views, and right ventricle (RV) focused four chamber were obtained with a frame rate between 50–70 frames/ second to calculate the global longitudinal strain (GLS). The GLS was quantified using the semiautomatic analysis GE Healthcare, Vingmed Ultrasound, Horten, Norway, software EchoPAC version 20.3. The normal reference value for GLS based on current recommendations was −18% [28].

### 2.3. Myocardial Work (MW)

MW was obtained from the pressure-strain loop area constructed from the LV pressure curves and GLS, as recommended by current guidelines [29,30,31]. The MW parameters acquired using GE Healthcare, Vingmed Ultrasound, Horten, Norway, EchoPAC software, version 20.3, were global work index (GWI), global work efficiency (GWE), global wasted work (GWW), and global constructive work (GCW). According to the NORRE study, the normal reference values for the MW parameters are GWI 1896 ± 308 mmHg%, GCW mmHg% 2232 ± 331, GWW mmHg% 78.5 (53–122.2), and GWE % 96 (94–97) [32].

### 2.4. Statistical Analysis

Descriptive statistics were performed for all the study variables. Continuous variables were expressed as a mean ± standard deviation (SD) or median [interquartile (IQR)] for skewed variables. Categorical variables were expressed as percentages. The normality of the data was tested using Kolmogorov-Smirnov test. Comparison of the continuous variables was performed using Student’s *t*-test or Mann-Whitney U-test and of binominal variables using chi-square or Fisher’s exact test, respectively. Univariable and multivariable logistic regression models with backward selection were used to evaluate the potential predictors of persistent dyspnea in COVID-19-recovered patients. The variables included in the statistical model were tested for collinearity using linear regression analysis with a variance inflation factor (VIF) between 1 and 10.

The data were analyzed using IBM SPSS Statistic for Windows, Version 26.0 (IBM Corp., Armonk, NY, USA). A *p* < 0.05 was considered significant.

## 3. Results

A total of 66 patients (mean age 51.3 ± 11.1 years, 45 (68.2%) males) were included in the final analysis. Of those, 23 (34.8%) patients reported dyspnea at one-year. Baseline characteristics including clinical data, laboratory values, spirometry and chest CT parameters are available in Supplementary Materials Table S1.

**Figure 2:**
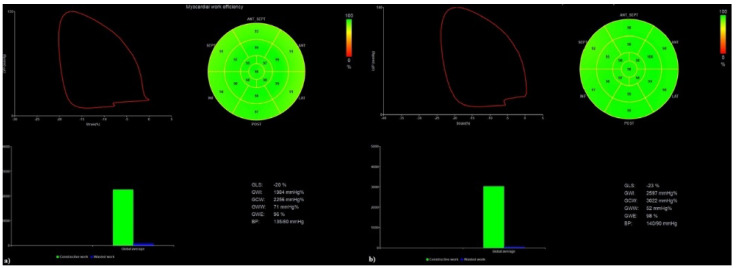
Myocardial work analysis in recovered COVID-19 patients.

In summary, the following parameters were significantly different in patients with and without persistent dyspnea at the one-year follow-up: GLS LV (−19.9 ± 2.1 versus (vs.) −21.3 ± 2.3, *p* = 0.039); GCW (2183.7 ± 487.9 vs. 2483.1 ± 422.4, *p* = 0.024); and GWI (1960.0 ± 396.2 vs. 2221.1 ± 407.9, *p* = 0.030) (Figure 2).

Figure 2 shows an example of the MW measurements' differences between a recovered COVID-19 patient without history of cardiorespiratory diseases but with persistent dyspnea at the one-year follow-up, where TTE evaluation showed left ventricle GLS of -20%, with GWI of 1984 mmHg% and GCW of 2256 mmHg% (a) and recovered COVID-19 patient without history of cardiorespiratory diseases and without persistent dyspnea at the one-year follow-up, where TTE evaluation showed left ventricle GLS of -23%, with GWI of 2597 mmHg% and GCW of 3022 mmHg% (b).

Detailed data on the LV and RV TTE parameters' measurements are available in Table 1.

### Predictors of Persistent Dyspnea

Multivariable logistic regression showed that GCW and GWI were inversely and independently associated with persistent dyspnea one-year after COVID-19 infection (*p =* 0.035, OR 0.998, 95% CI 0.997–1.000; *p* = 0.040, OR 0.998, 95% CI 0.997–1.000) (Table 2).

Univariable logistic regression for persistent dyspnea at one year follow-up is available in Supplementary Materials Table S2.

## 4. Discussion

The presence of persistent dyspnea in one third of the recovered COVID-19 patients without previous history of cardiac or respiratory diseases at one-year follow-up and its association with myocardial performance, assessed using MW, represent the core findings of our study.

In the present study, 34.8% of the recovered COVID-19 patients without pre-existing cardio-respiratory diseases presented dyspnea at one-year follow-up, in line with current data on long COVID [16,33].

Moreover, earlier reports on recovered patients following the infection with severe acute respiratory syndrome coronavirus (SARS) and the Middle East respiratory syndrome coronavirus (MERS-CoV) attributed the presence of dyspnea to functional respiratory impairment and radiological abnormalities, which were present in more than a third of the patients [34,35]. Although there are several similarities between COVID-19 and previous SARS and MERS epidemics, evidence regarding possible pulmonary sequelae in recovered COVID-19 patients is still scarce. In the present study, 27.3% of the patients had residual mat glass opacities on chest CT at six months follow-up, and 10.6% of the patients developed pulmonary fibrosis. Other studies reported fibrotic-like changes within the lung during the first six months from the disease onset in up to 43.2% recovered COVID-19 patients, emphasizing the persistence of pulmonary parenchymal abnormalities after the acute phase of COVID-19 [36,37]. However, the presence of abnormalities on follow-up chest CTs was not significantly correlated with symptoms in either earlier radiological reports or in the current study.

A potential cause of dyspnea in recovered COVID-19 patients could be represented by cardiac abnormalities, such as myocarditis-like patterns or ischemic injury, which were observed in more than half of the discharged patients at one to two months follow-up [38,39]. Nevertheless, these changes had little impact on the LVEF, [33,38,39,40], being related to the ongoing inflammation or pre-existing silent cardiac diseases. Moreover, most of the studies on long COVID had heterogeneous populations without a clear distinction between those with and without previous cardiac or respiratory comorbidities.

The present study focused on the one-year follow-up of patients without established cardiac or respiratory pathologies prior to COVID-19. In line with earlier COVID-19 follow-up reports [38,39], standard TTE parameters revealed a preserved global LV function irrespective of the presence of dyspnea at one year. However, LV myocardial performance assessed using MW was independently associated with persistent dyspnea. MW has recently emerged as a promising technique which can provide a more precise evaluation of the LV function by incorporating the LV pressure-strain relationship [29,30]. Similarly, in a retrospective study on hospitalized COVID-19 patients, a decrease in MW efficiency was associated with inflammation burden and in-hospital mortality [41]. Other small reports also showed reduced GWI in the presence of normal LVEF and GLS during the acute setting of the COVID-19 with an improved near to normal GWI after one month [42].

The applicability of MW in various cardiac pathologies has been suggested by several recent publications due to its lower load-dependency and hence higher sensitivity in detecting subclinical dysfunction than LVEF and GLS alone [29,31,43].

The early detection of subclinical cardiac abnormalities could contribute to a better understanding of the extent of myocardial damage following COVID-19 which could lead to persistent dyspnea at mid- and long- term follow-up. The findings of the present study emphasize the potential incremental value of MW compared to standard TTE parameters to detect subclinical cardiac dysfunction in COVID-19 [42].

Both GCW and GWI were inversely and independently associated with the persistence of dyspnea. Additionally, there was no relationship between the severity of the acute setting of the disease and dyspnea at the follow-up. Therefore, persistent dyspnea in patients without pre-existing cardiac or respiratory diseases, following COVID-19 might be attributed to subclinical cardiac dysfunction developed during the disease as suggested by the MW parameters.

These findings show that MW might represent a new tool for early identification of subclinical dysfunction in recovered COVID-19 patients who might need long-term cardiac follow-up.

### Study Limitations

This study has several limitations. This is a single centre study with a limited number of recovered COVID-19 patients. Echocardiography during hospitalization was performed only in selected cases due to logistical restrictions at the beginning of the pandemic. However, we focused our research on patients without pre-existing cardiac comorbidities. The cardiac biomarkers were not systematically performed at one-year follow-up; therefore, they were not included in the analysis. Multicentric prospective studies including various COVID-19 strains and the impact of vaccination are necessary to confirm our results regarding the long-term evolution of recovered patients.

## 5. Conclusions

Persistent dyspnea one-year after COVID-19 was present in more than a third of patients without known cardiovascular or pulmonary diseases. GCW and GWI were the only echocardiographic parameters independently associated with symptoms, suggesting a decrease in myocardial performance in this population and subclinical cardiac dysfunction.

## Figures and Tables

**Table 1:** Echocardiographic parameters.

Echocardiographic Parameters	Total Population (*n* = 66)	Dyspnea+ (*n* = 23)	Dyspne− (*n* = 43)	*p* Value
LVEF (%)	56.9 ± 4.6	55.7 ± 4.6	57.6 ± 4.5	0.131
LA volume (mL/m^2^)	27.1 ± 7.5	27.1 ± 9.9	27.1 ± 6.0	0.986
FAC (%)	41.2 ± 8.8	40.8 ± 7.4	41.3 ± 9.6	0.838
TAPSE (mm)	24.8 ± 4.3	24.5 ± 4.9	25.0 ± 4.0	0.672
E/A	0.9 ± 0.2	1.0 ± 0.2	1.1 ± 0.3	0.046
E/E’	9.1 ± 3.1	7.8 ± 2.4	7.2 ± 2.1	0.323
S’ (cm/s)	12.9 ± 2.1	12.3 ± 2.3	13.3 ± 1.8	0.077
PAP (mmHg)	16.5 ± 14.5	17.9 ± 14.1	15.7 ± 14.8	0.562
GLS LV (%)	−20.9 ± 2.3	−19.9 ± 2.1	−21.3 ± 2.3	0.039
GCW (mmHg%)	2381.4 ± 463.6	2183.7 ± 487.9	2483.1 ± 422.4	0.024
GWW (mmHg%)	64.9 ± 33.1	60.9 ± 34.4	67.0 ± 32.7	0.530
GWE (%)	96.8 ± 2.5	96.4 ± 1.7	97.0 ± 2.8	0.425
GWI (mmHg%)	2132.5 ± 419.2	1960.0 ± 396.2	2221.1 ± 407.9	0.030
GLS RV (%)	−21.9 ± 3.2	−21.3 ± 2.9	−22.3 ± 3.4	0.375
GLS RV free wall (%)	−24.4 ± 4.2	−25.2 ± 3.0	−25.5 ± 4.8	0.831
LV echocardiographic abnormalities in 66 patients				
LV systolic global dysfunction (*n*,%)	1 (1.6%)	1 (4.8%)	0 (0%)	0.181
LV type 1 diastolic dysfunction (*n*,%)	11 (16.7%)	3 (14.3%)	8 (21.6%)	0.493
LV type 2 diastolic dysfunction (*n*,%)	1 (1.5%)	1 (4.8%)	0 (0%)	0.181

LVEF-left ventricle ejection fraction; LA-left atrium, FAC-fractional area change; TAPSE-tricuspid annular plane systolic excursion; PAP-pulmonary artery pressure; GLS-global longitudinal strain; GCW-global constructive work; GWW-global wasted work; GWE-global work efficiency; GWI-global work index; RV-right ventricle.

**Table 2:** Predictors of persistent dyspnea in COVID-19-recovered patients.

Parameter	Univariable Analysis			Multivariable Analysis					
	OR	95% CI	*p* Value	OR	95% CI	*p* Value	OR	95% CI	*p* Value
GLS LV	1.321	1.004–1.738	0.047	1.171	0.848–1.616	0.338	1.179	0.853–1.628	0.318
GCW	0.998	0.997–1.000	0.035	0.998	0.997–1.000	0.035			
GWI	0.998	0.997–1.000	0.040				0.998	0.997–1.000	0.040

GLS—global longitudinal work; LV—left ventricle; GCW—global constructive work; GWI—global work index.

## Data Availability

The data presented in this study are available on request from the corresponding author.

## Supplementary Materials

The following are available online at https://www.mdpi.com/article/10.3390/diagnostics12010057/s1, Table S1: Baseline characteristics, Table S2: Univariable analysis for persistent dyspnea one year following COVID-19.

## References

[1] Nishiga M., Wang D.W., Han Y., Lewis D.B., Wu J.C. (2020). COVID-19 and cardiovascular disease: From basic mechanisms to clinical perspectives. Nat. Rev. Cardiol..

[2] Xu H., Hou K., Xu R., Li Z., Fu H., Wen L., Xie L., Liu H., Selvanayagam J.B., Zhang N. (2020). Clinical Characteristics and Risk Factors of Cardiac Involvement in COVID-19. J. Am. Heart Assoc..

[3] WHO (2021). COVID-19 Weekly Epidemiological Update.

[4] Pesaresi M., Pirani F., Tagliabracci A., Valsecchi M., Procopio A.D., Busardò F.P., Graciotti L. (2020). SARS-CoV-2 identification in lungs, heart and kidney specimens by transmission and scanning electron microscopy. Eur. Rev. Med Pharmacol. Sci..

[5] Núñez-Gil I.J.J., Fernández-Ortiz A., Eid C.M., Huang J., Romero R., Becerra-Muñoz V.M., Uribarri A., Feltes G., Trabatoni D., Fernandez-Rozas I. (2021). Underlying heart diseases and acute COVID-19 outcomes. Cardiol. J..

[6] He X.W., Lai J.S., Cheng J., Wang M.W., Liu Y.J., Xiao Z.C., Xu C., Li S.S., Zeng H.S. (2020). Impact of complicated myocardial injury on the clinical outcome of severe or critically ill COVID-19 patients. Zhonghua Xin Xue Guan Bing Za Zhi.

[7] Grant M.C., Geoghegan L., Arbyn M., Mohammed Z., McGuinness L., Clarke E.L., Wade R.G. (2020). The prevalence of symptoms in 24,410 adults infected by the novel coronavirus (SARS-CoV-2; COVID-19): A systematic review and meta-analysis of 148 studies from 9 countries. PLoS ONE.

[8] Li X., Guan B., Su T., Liu W., Chen M., Waleed K.B., Guan X., Gary T., Zhu Z. (2020). Impact of cardiovascular disease and cardiac injury on in-hospital mortality in patients with COVID-19: A systematic review and meta-analysis. Heart.

[9] Hamming I., Timens W., Bulthuis M.L.C., Lely A.T., Navis G.J., van Goor H. (2004). Tissue distribution of ACE2 protein, the functional receptor for SARS coronavirus. A first step in understanding SARS pathogenesis. J. Pathol..

[10] Siripanthong B., Nazarian S., Muser D., Deo R., Santangeli P., Khanji M.Y., Cooper L.T., Chahal C.A.A. (2020). Recognizing COVID-19–related myocarditis: The possible pathophysiology and proposed guideline for diagnosis and management. Heart Rhythm..

[11] Lala A., Johnson K.W., Januzzi J.L., Russak A.J., Paranjpe I., Richter F., Zhao S., Somani S., Van Vleck T., Vaid A. (2020). Prevalence and Impact of Myocardial Injury in Patients Hospitalized With COVID-19 Infection. J. Am. Coll. Cardiol..

[12] Deng Q., Hu B., Zhang Y., Wang H., Zhou X., Hu W., Cheng Y., Yan J., Ping H., Zhou Q. (2020). Suspected myocardial injury in patients with COVID-19: Evidence from front-line clinical observation in Wuhan, China. Int. J. Cardiol..

[13] Messina A., Sanfilippo F., Milani A., Calabrò L., Negri K., García M.I.M., Astuto M., Vieillard-Baron A., Cecconi M. (2021). COVID-19-related echocardiographic patterns of cardiovascular dysfunction in critically ill patients: A systematic review of the current literature. J. Crit. Care.

[14] Barman H.A., Atici A., Tekin E.A., Baycan O.F., Alici G., Meric B.K., Sit O., Genc O., Er F., Gungor B. (2021). Echocardiographic features of patients with COVID-19 infection: A cross-sectional study. Int. J. Cardiovasc. Imag..

[15] Mahmoud-Elsayed H.M., Moody W.E., Bradlow W.M., Khan-Kheil A.M., Hudsmith L.E., Steeds R.P. (2020). Echocardiographic Findings in Covid-19 Pneumonia. Can. J. Cardiol..

[16] Varghese J., Sandmann S., Ochs K., Schrempf I.-M., Frömmel C., Dugas M., Schmidt H.H., Vollenberg R., Tepasse P.-R. (2021). Persistent symptoms and lab abnormalities in patients who recovered from COVID-19. Sci. Rep..

[17] Moulson N., Petek B.J., Drezner J.A., Harmon K.G., Kliethermes S.A., Patel M.R., Baggish A.L., Asif I.M., Borchers J., Edenfield K.M. (2021). SARS-CoV-2 Cardiac Involvement in Young Competitive Athletes. Circulation.

[18] Brito D., Meester S., Yanamala N., Patel H.B., Balcik B.J., Casaclang-Verzosa G., Seetharam K., Riveros D., Beto R.J., Balla S. (2021). High Prevalence of Pericardial Involvement in College Student Athletes Recovering From COVID-19. JACC: Cardiovasc. Imag..

[19] Ricci F., Aung N., Gallina S., Zemrak F., Fung K., Bisaccia G., Paiva J.M., Khanji M.Y., Mantini C., Palermi S. (2021). Cardiovascular magnetic resonance reference values of mitral and tricuspid annular dimensions: The UK Biobank cohort. J. Cardiovasc. Magn. Reson..

[20] Palermi S., Serio A., Vecchiato M., Sirico F., Gambardella F., Ricci F., Iodice F., Radmilovic J., Russo V., D’Andrea A. (2021). Potential role of an athlete-focused echocardiogram in sports eligibility. World J. Cardiol..

[21] Puntmann V.O., Carerj M.L., Wieters I., Fahim M., Arendt C., Hoffmann J., Shchendrygina A., Escher F., Vasa-Nicotera M., Zeiher A.M. (2020). Outcomes of Cardiovascular Magnetic Resonance Imaging in Patients Recently Recovered From Coronavirus Disease 2019 (COVID-19). JAMA Cardiol..

[22] Daniels C.J., Rajpal S., Greenshields J.T., Rosenthal G.L., Chung E.H., Terrin M., Jeudy J., Mattson S.E., Law I.H., Borchers J. (2021). Prevalence of Clinical and Subclinical Myocarditis in Competitive Athletes with Recent SARS-CoV-2 Infection. JAMA Cardiol..

[23] Fayol A., Livrozet M., Boutouyrie P., Khettab H., Betton M., Tea V., Blanchard A., Bruno R., Hulot J. (2021). French COVID cohort study group Cardiac performance in patients hospitalized with COVID-19: A 6 month follow-up study. ESC Heart Fail..

[24] Daher A., Balfanz P., Cornelissen C., Müller A., Bergs I., Marx N., Müller-Wieland D., Hartmann B., Dreher M., Müller T. (2020). Follow up of patients with severe coronavirus disease 2019 (COVID-19): Pulmonary and extrapulmonary disease sequelae. Respir. Med..

[25] Moody W.E., Liu B., Mahmoud-Elsayed H.M., Senior J., Lalla S.S., Khan-Kheil A.M., Brown S., Saif A., Moss A., Bradlow W.M. (2021). Persisting Adverse Ventricular Remodeling in COVID-19 Survivors: A Longitudinal Echocardiographic Study. J. Am. Soc. Echocardiogr..

[26] Lancellotti P., Zamorano J.L., Habib G., Badano L. (2016). The EACVI Textbook of Echocardiography.

[27] Lang R.M., Badano L.P., Mor-Avi V., Afilalo J., Armstrong A., Ernande L., Flachskampf F.A., Foster E., Goldstein S.A., Kuznetsova T. (2015). Recommendations for Cardiac Chamber Quantification by Echocardiography in Adults: An Update from the American Society of Echocardiography and the European Association of Cardiovascular Imaging. J. Am. Soc. Echocardiogr..

[28] Farsalinos K.E., Daraban A.M., Ünlü S., Thomas J.D., Badano L., Voigt J.-U. (2015). Head-to-Head Comparison of Global Longitudinal Strain Measurements among Nine Different Vendors. J. Am. Soc. Echocardiogr..

[29] Papadopoulos K., Özden Tok Ö., Mitrousi K., Ikonomidis I. (2021). Myocardial Work: Methodology and Clinical Applications. Diagnostics.

[30] Russell K., Eriksen M., Aaberge L., Wilhelmsen N., Skulstad H., Remme E.W., Haugaa K.H., Opdahl A., Fjeld J.G., Gjesdal O. (2012). A novel clinical method for quantification of regional left ventricular pressure–strain loop area: A non-invasive index of myocardial work. Eur. Heart J..

[31] Russell K., Eriksen M., Aaberge L., Wilhelmsen N., Skulstad H., Gjesdal O., Edvardsen T., Smiseth O.A. (2013). Assessment of wasted myocardial work: A novel method to quantify energy loss due to uncoordinated left ventricular contractions. Am. J. Physiol. Heart Circ. Physiol..

[32] Manganaro R., Marchetta S., Dulgheru R., Ilardi F., Sugimoto T., Robinet S., Cimino S., Go Y.Y., Bernard A., Kacharava G. (2019). Echocardiographic reference ranges for normal non-invasive myocardial work indices: Results from the EACVI NORRE study. Eur. Heart J. Cardiovasc. Imag..

[33] Maestrini V., Birtolo L.I., Francone M., Galardo G., Galea N., Severino P., Alessandri F., Colaiacomo M.C., Cundari G., Chimenti C. (2021). Cardiac involvement in consecutive unselected hospitalized COVID-19 population: In-hospital evaluation and one-year follow-up. Int. J. Cardiol..

[34] Ahmed H., Patel K., Greenwood D., Halpin S., Lewthwaite P., Salawu A., Eyre L., Breen A., O’Connor R., Jones A. (2020). Long-term clinical outcomes in survivors of severe acute respiratory syndrome and Middle East respiratory syndrome coronavirus outbreaks after hospitalisation or ICU admission: A systematic review and meta-analysis. J. Rehabil. Med..

[35] Madjid M., Safavi-Naeini P., Solomon S.D., Vardeny O. (2020). Potential Effects of Coronaviruses on the Cardiovascular System: A Review. JAMA Cardiol..

[36] Parry A.H., Wani A.H., Shah N.N., Jehangir M. (2021). Medium-term chest computed tomography (CT) follow-up of COVID-19 pneumonia patients after recovery to assess the rate of resolution and determine the potential predictors of persistent lung changes. Egypt. J. Radiol. Nucl. Med..

[37] Han X., Fan Y., Alwalid O., Li N., Jia X., Yuan M., Li Y., Cao Y., Gu J., Wu H. (2021). Six-month Follow-up Chest CT Findings after Severe COVID-19 Pneumonia. Radiol..

[38] Özer S., Candan L., Özyıldız A.G., Turan O.E. (2021). Evaluation of left ventricular global functions with speckle tracking echocardiography in patients recovered from COVID-19. Int. J. Cardiovasc. Imag..

[39] Kotecha T., Knight D.S., Razvi Y., Kumar K., Vimalesvaran K., Thornton G., Patel R., Chacko L., Brown J.T., Coyle C. (2021). Patterns of myocardial injury in recovered troponin-positive COVID-19 patients assessed by cardiovascular magnetic resonance. Eur. Heart J..

[40] Heuvel F.M.A.V.D., Vos J.L., Koop Y., van Dijk A.P.J., Duijnhouwer A.L., de Mast Q., van de Veerdonk F.L., Bosch F., Kok B., Netea M.G. (2020). Cardiac function in relation to myocardial injury in hospitalised patients with COVID-19. Neth. Heart J..

[41] Minhas A.S., Gilotra N.A., Goerlich E., Metkus T., Garibaldi B.T., Sharma G., Bavaro N., Phillip S., Michos E.D., Hays A.G. (2021). Myocardial Work Efficiency, A Novel Measure of Myocardial Dysfunction, Is Reduced in COVID-19 Patients and Associated With In-Hospital Mortality. Front. Cardiovasc. Med..

[42] Jaglan A., Roemer S., Jan M.F., Khandheria B.K. (2021). Myocardial work index: A glimmer of hope in COVID-19. Eur. Heart J. Cardiovasc. Imag..

[43] Hubert A., Le Rolle V., Leclercq C., Galli E., Samset E., Casset C., Mabo P., Hernandez A., Donal E. (2018). Estimation of myocardial work from pressure–strain loops analysis: An experimental evaluation. Eur. Heart J. Cardiovasc. Imaging.

