# Supplementary material for: Subclinical Myocardial Dysfunction in Patients with Persistent Dyspnea One Year after COVID-19
Source: Diagnostics (Basel). 2021 Dec 28;12(1):57. doi: 10.3390/diagnostics12010057 (PMC8775030; doi:10.3390/diagnostics12010057)
Supplement: Supplementary file 1 [file diagnostics-12-00057-s001.zip › diagnostics-1512092-supplementary.pdf]

Table S1. Baseline characteristics

Demographics	Total population (n=66)	Dyspnoea + (n=23)	Dyspnoea - (n=43)	Ppvalue
Age (years)	51.3±11.1	51.0±11.3	51.6±11.1	0.836
Gender (male, %)	45(68.2%)	14(60.9%)	31(72.1%)	0.351
BMI (kg/m^2)	27.6±4.7	28.5±6.2	27.2±3.6	0.292
**History**				
Dyslipidaemia (n, %)	13(19.7%)	4(17.4%)	9(20.9%)	0.731
Diabetes mellitus type 2 (n, %)	11(16.7%)	3(13%)	8(18.6%)	0.564
Obesity (n, %)	17(25.8%)	7(30.4%)	10(23.3%)	0.525
Smoking (n, %)	8(12.1%)	2(8.7%)	6(14%)	0.533
Cancer (n, %)	2(3%)	1(4.3%)	1(2.3%)	0.648
Chronic autoimmune disease (n, %)	4(6.1%)	2(8.7%)	2(4.7%)	0.512
**Clinical characteristics at admission**				
Systolic blood pressure (mmHg)	127.5±15.6	129.3±13.9	126.5±16.5	0.497
Diastolic blood pressure (mmHg)	79.3±12.6	84.1±12.8	76.7±11.8	0.022
Heart Rate (bpm)	95.5±19.0	101.2±15.8	92.4±20.1	0.075
Respiratory rate	26.2±13.5	29.7±20.8	24.2±6.6	0.273
Temperature (ºC)	37.5±1.0	37.9±1.1	37.2±0.9	0.021
SpO_2 (%)	94.9±4.0	94.8±3.7	94.9±4.2	0.900
ICU admission (n,%)	12(18.2%)	3(13%)	9(20.9%)	0.429
**Laboratory findings at admission**				
Haemoglobin (g/dl)	14.1±1.4	14.1±1.5	14.1±1.4	0.945
WBC (10^3/mm^3)	7.1±2.8	7.4±2.6	7.0±2.9	0.596
NL ratio	6.1±4.1	6.3±4.5	6.0±4.0	0.794

CRP (mg/L)	130.9±88.6	145.1±83.0	123.3±91.4	0.344
Troponin T (u/L)	0.010±0.008	0.010±0.005	0.009±0.009	0.891
Ferritin (μg/L)	1215.0±1539.4	1528.1±2125.9	969.0±866.2	0.379
D – dimer (ng/ ml)	1045.6±1854.2	670.3±564.9	1260.1±2278.7	0.388
INR	1.1±0.1	1.1±0.1	1.1±0.1	0.582
LDH (U/L)	868.8±333.4	891.4±375.1	857.7±315.2	0.707
Creatinine (mg/dL)	0.8±0.2	0.8±0.2	0.8±0.2	0.306
Lactate (mEg/L)	1.0±0.4	1.1±0.4	0.9±0.4	0.120
**Physical examinations at one year FU**				
Systolic blood pressure (mmHg)	130.4±18.0	127.7±14.2	131.9±19.8	0.380
Diastolic blood pressure(mmHg)	79.1±12.1	77.7±13.2	79.9±11.6	0.504
Heart Rate (bpm)	72.6±10.6	74.6±9.4	71.5±11.2	0.306
**Spirometry at 6 months**				
Normal lung function (n,%)	31(47%)	7(30.4%)	24(55.8%)	0.049
Restriction pattern (n,%)	19(28.8%)	9 (39.1%)	10(23.3%)	0.175
Obstructive pattern (n,%)	3(4.5%)	1(4.3%)	2(4.7%)	0.955
**Spirometry at 12 months**				
Normal lung function (n,%)	17(25.8%)	3(13%)	14(32.6%)	0.084
Restriction pattern (n,%)	8(12.1%)	4(17.4%)	4(9.3%)	0.337
Obstructive pattern (n,%)	3(4.5%)	1(4.3%)	2(4.7%)	0.955
**Computed tomography**				
Fibrosis (n,%)	7(10.6%)	2(8.7%)	5 (11.6%)	0.712
Residual ground glass opacity (n,%)	18(27.3%)	3(13%)	15(34.9%)	0.058
Total length of hospitalization (days)	12.0±10.7	11.4±10.5	11.9±10.9	0.857

Duration till follow-up (days)	343.8±34.6	346.9±27.0	342.2±38.3	0.603

BMI- body mass index; SpO2 - peripheral oxygen saturation; WBC - white blood cells; NLR-neutrophil lymphocyte ratio; CRP- C reactive protein; LDH - lactate dehydrogenase; FU- follow up; ICU- intensive care unit

Table S2. Univariable analysis for persistent dyspnea one year following COVID-19

Table 2			
Parameter	Univariable analysis		
	OR	95% CI	P value
Age	0.995	0.951-1.042	0.833
Gender (male)	0.602	0.207-1.756	0.353
Diastolic blood pressure	1.055	1.004-1.108	0.033
Temperature	1.848	1.078-3.169	0.026
E/A	0.082	0.007-1.030	0.053
LV GLS	1.321	1.004-1.738	0.047
GCW	0.998	0.997-1.000	0.035
GWI	0.998	0.997-1.000	0.040
Normal lung function at 6 months spirometry	0.346	0.118-0.346	0.053

LV GLS - left ventricle global longitudinal work; GCW- global constructive work; GWI-global work index
